# Supplementary material for: Decreasing pdzd8-mediated mito–ER contacts improves organismal fitness and mitigates Aβ42 toxicity
Source: Life Sci Alliance. 2022 Jul 13;5(11):e202201531. doi: 10.26508/lsa.202201531 (PMC9279675; doi:10.26508/lsa.202201531)
Supplement: Supplementary file 1 [file LSA-2022-01531_TableS1.docx]

## Table S1: List of fly strains used in this study

| **Label** | **Line** | **Source** | **Reference** | **RRID** |
| --- | --- | --- | --- | --- |
| nSyb | P{nSyb-GAL4.S}3 | BL51635 | Bloomington | BDSC_51635 |
| OK371 | P{GawB}VGlut^OK371^ | BL26160 | ([Mahr & Aberle, 2006](#_ENREF_44)) | BDSC_26160 |
| da | da-GAL4 | BL55850 | Bloomington | BDSC_55850 |
| Act | P{Act5C-GAL4}25FO1 | BL4414 | Bloomington | BDSC_4414 |
| CCAP | P{CCAP-GAL4}16 | BL25685 | ([Park *et al.*, 2003](#_ENREF_55)) | BDSC_25685 |
| tether | UAS-tether | Scorrano Lab | ([Basso *et al.*, 2018](#_ENREF_6)) tether high (TH) construct from ([Csordas *et al.*, 2006](#_ENREF_11)) |  |
| *LacZ*-RNAi | P{GD936}v51446 II  UAS-LacZ-RNAi | VDRC | ([Dietzl *et al*, 2007](#_ENREF_18)) | FlyBase_FBst0469426 |
| *Luc*-RNAi | M{UAS-Ren/Luc}ZH-51C | BL64774 | Bloomington | BDSC_64774 |
| *pdzd8*-RNAi | NIG 10362R-2  UAS-pdzd8-RNAi III | NIG-Fly | The fly stock was obtained from NIG-Fly Stock Center. |  |
| mitoGFP (II) | P{UAS-mito-HA-GFP.AP}2 | BL8442 | ([Pilling *et al*, 2006](#_ENREF_57)) | BDSC_8442 |
| mitoGFP (III) | P{UAS-mito-HA-GFP.AP}3 | BL8443 | ([Pilling *et al.*, 2006](#_ENREF_57)) | BDSC_8443 |
| mitoCherry | P{UAS-mCherry.mito.OMM}3 | Alessio Vagnoni | ([Vagnoni *et al*, 2016](#_ENREF_73)) | BDSC_66533 |
| ER-Tom | PBac{20XUAS-tdTomato-Sec61β}VK00037 | BL64746 | ([Summerville *et al*, 2016](#_ENREF_67)) | BDSC_64746 |
| SPLICS | SPLICSs#4 | This study | Construct from ([Cieri *et al.*, 2017](#_ENREF_9)) |  |
| mitoQC | UAS-mitoQC (II) | Whitworth Lab | ([Lee *et al.*, 2018b](#_ENREF_40)) | BDSC_91640 |
| Aβ_42_ | P{UAS-Aβ_42_.Arctic} | Isabel Palacios | ([Crowther *et al.*, 2005](#_ENREF_10)) FBal0248069 |  |
| *pdzd8*-HA | P{UAS-pdzd8-HA}attP40 | This study | University of Cambridge Department of Genetics Fly Facility |  |

References

Dietzl G, Chen D, Schnorrer F, Su KC, Barinova Y, Fellner M, Gasser B, Kinsey K, Oppel S, Scheiblauer S, et al (2007) A genome-wide transgenic RNAi library for conditional gene inactivation in Drosophila. Nature 448: 151–156. 10.1038/nature05954

Mahr A, Aberle H (2006) The expression pattern of the *Drosophila* vesicular glutamate transporter: A marker protein for motoneurons and glutamatergic centers in the brain. Gene Expr Patterns 6: 299–309. 10.1016/j.modgep.2005.07.006

Pilling AD, Horiuchi D, Lively CM, Saxton WM (2006) Kinesin-1 and Dynein are the primary motors for fast transport of mitochondria in *Drosophila* motor axons. Mol Biol Cell 17: 2057–2068. 10.1091/mbc.e05-06-0526

Summerville JB, Faust JF, Fan E, Pendin D, Daga A, Formella J, Stern M, McNew JA (2016) The effects of ER morphology on synaptic structure and function in *Drosophila melanogaster*. J Cell Sci 129: 1635–1648. 10.1242/jcs.184929

Vagnoni A, Hoffmann PC, Bullock SL (2016) Reducing Lissencephaly-1 levels augments mitochondrial transport and has a protective effect in adult *Drosophila* neurons. J Cell Sci 129: 178–190. 10.1242/jcs.179184
