# Supplementary material for: Decreasing pdzd8-mediated mito–ER contacts improves organismal fitness and mitigates Aβ42 toxicity
Source: Life Sci Alliance. 2022 Jul 13;5(11):e202201531. doi: 10.26508/lsa.202201531 (PMC9279675; doi:10.26508/lsa.202201531)
Supplement: Supplementary file 2 [file LSA-2022-01531_TableS2.docx]

## Table S2: Genotypes in figures

| Figure 1 (B, C) | *LacZ*-RNAi/+; nSyb/+ |
| --- | --- |
|  | *pdzd8*-RNAi/nSyb |
| Figure 1 (D, E) | SPLICS/*LacZ*-RNAi; nSyb/+ |
|  | SPLICS/+; *pdzd8*-RNAi/nSyb |
| Figure 1 (F, G, H) | ER-Tom, mitoGFP/ *LacZ*-RNAi; da/+ |
|  | ER-Tom, mitoGFP/+; da/*pdzd8*-RNAi |
| Figure 2 (A, C) | *LacZ*-RNAi/+; nSyb/+ |
|  | *pdzd8*-RNAi/nSyb |
| Figure 2 (B, D) | *LacZ*-RNAi/+; nSyb/+ |
|  | tether/nSyb |
| Figure 3 (A-D) | *LacZ*-RNAi/+; nSyb/+ |
|  | *pdzd8*-RNAi/nSyb |
| Figure 4 (A-E) | CCAP/+; mitoGFP/+ |
|  | CCAP/+; *pdzd8*-RNAi/mitoGFP |
|  | CCAP/+; tether/mitoGFP |
| Figure 4 (F-I) | OK371, mitoGFP/*LacZ*-RNAi |
|  | OK371, mitoGFP/+; *pdzd8*-RNAi/+ |
| Figure 5 (A-F) | mitoQC/*LacZ*-RNAi; nSyb/+ |
|  | mitoQC/+; nSyb/*pdzd8*-RNAi |
| Figure 6 (A-B) | *LacZ*-RNAi/SPLICS; nSyb/+ |
|  | Aβ_42_/SPLICS; nSyb/+ |
| Figure 6 (C-H) | *LacZ*-RNAi/+; nSyb/+, |
|  | *LacZ*-RNAi/Aβ_42_; nSyb/+ |
|  | Aβ_42_/+; *pdzd8*-RNAi/nSyb |
| Figure S1 (C) | Act/*LacZ*-RNAi (females) |
|  | Act/+; *pdzd8*-RNAi/+ (females) |
| Figure S1 (D) | *LacZ*-RNAi/+; nSyb/+ |
|  | *pdzd8*-RNAi/nSyb |
| Figure S1 (E) | ER-Tom, mitoGFP/*LacZ*-RNAi; nSyb/+ |
|  | ER-Tom, mitoGFP/+; nSyb/*pdzd8*-RNAi |
| Figure S2 (B) | SPLICSs/+; nSyb-GAL4/+ |
| Figure S2 (D) | SPLICS/*LacZ*-RNAi; nSyb/+ |
|  | SPLICS/+; tether/nSyb |
| Figure S3 | mitoGFP/+; nSyb/+ |
|  | nSyb/*Luciferase*-RNAi |
|  | *LacZ*-RNAi/+; nSyb/+ |
|  | *pdzd8*-RNAi/nSyb |
| Figure S4 (A) | OK371/*LacZ*-RNAi |
|  | OK371/+; *pdzd8*-RNAi/+ |
| Figure S4 (B) | *LacZ*-RNAi/+; mitoCherry/nSyb |
|  | *LacZ*-RNAi/*pdzd8*-HA; nSyb/+ |
|  | *pdzd8*-HA/+; *pdzd8*-RNAi/nSyb |
|  | tether/nSyb |
| Figure S4 (C) | tether/nSyb (all) |
| Figure S4 (D) | *LacZ*-RNAi/+; nSyb/+ |
|  | *pdzd8*-RNAi/nSyb |
